# Comparing the Effect of Monofocal and Multifocal Intraocular Lenses on Macular Surgery

**DOI:** 10.1155/2020/1375298

**Published:** 2020-07-20

**Authors:** A. Altun

**Affiliations:** Bahcesehir University, Faculty of Medicine, Department of Ophthalmology, Istanbul, Turkey

## Abstract

**Aim:**

To compare the effects of previously implanted monofocal and multifocal intraocular lenses (IOL) on macular surgery.

**Methods:**

Seventy eyes of 70 patients with epiretinal membrane (ERM) and symptomatic vitromacular traction syndrome that previously had IOL implantation for cataract surgery were included in this prospective randomized clinical trial. Cases were divided into two groups. Group 1 and Group 2 were composed of eyes with monofocal and multifocal IOLs, respectively. The effects of refraction error and IOL decentration at the time of macular surgery performed for ERM and ILM peeling, according to the lens type, were investigated. Pars plana vitrectomy (PPV) was performed to peel ERM and ILM in all cases. Complete ophthalmological examination, fundus fluorescein angiography, and optical coherence tomography imaging were made to all cases, preoperatively and postoperatively.

**Results:**

The mean BCVA in Group 1 and Group 2 improved from 0.69 ± 0.15 and 0.38 ± 0.14 logMAR to 0.40 ± 0.14 and 0.10 ± 0.04 logMAR, respectively, at the 6^th^ month. There was no statistically significant difference between the groups in terms of the mean spherical refraction error (*P* > 0.05) and IOL decentration level (*P* > 0.05). The mean time required for macular surgery in Group 2 was statistically significantly longer than that for Group 1 (*P* < 0.05). There was no statistically significant relationship between IOL decentration and macular surgery time in Group 1 (*P* > 0.05), but there it was found in Group 2 (*P* < 0.05). In Group 2, there was a positive correlation between IOL decentration and macular surgery time.

**Conclusion:**

In cases with multifocal IOL implants, especially with lens decentration, the time of macular surgery for ERM and ILM peeling during PPV is longer than that of eyes with monofocal IOL due to fluctuations in the clarity of the surgeon's view.

## 1. Introduction

The ongoing development of intraocular lens (IOL) technology has led to significant improvements in refractive results. New and innovative ways to reach the desired refractive targets after the operation resume to develop continuously. As the popularity and availability of premium IOL increased, the goals of cataract surgery began to expand. Today, cataract surgery aims not only to provide vision rehabilitation but also to provide vision as far as possible, including intermediate and near vision. Multifocal IOLs serving these purposes could be classified as diffractive or refractive according to their designs [1, 2].

Multifocal IOL implantation is an important treatment option for presbyopia after cataract surgery. Many studies have reported that multifocal IOLs are more successful than near monofocals in near visual acuity, glasses independence rates, and patient satisfaction [2–4]. Unfortunately, multifocal IOLs may cause a decrease in contrast sensitivity and negative visual outcomes, such as halos and star bursts [5–7]. Tolerance to visual phenomena caused by multifocal IOLs usually improves over time. Researchers believe the brain adjusts to the altered visual input over time through neural adaptation [8].

Several contraindicated conditions for multifocal IOL implantation have been reported in the literature. Corneal pathologies, conditions that can cause IOL decentration or subluxation, asymmetric capsulorhexis, haptic deformation, diabetic retinopathy, age-related macular degeneration and epiretinal membrane, retinitis pigmentosa, and Stargardt's disease are some of the reported contraindications [9]. Contraindicated situations associated with artificial lens may develop after multifocal IOL implantation. In this randomized and controlled study, we aimed to investigate the effect of preimplanted monofocal or multifocal IOLs on macular surgery time.

## 2. Methods

Patients who presented to our clinic with symptomatic epiretinal membrane (ERM) and vitromacular traction syndrome, between January 2019 and January 2020, were included in this study. Macular pathology associated to ERM was defined by the OCT staging system. Stage 1, stage 2, stage 3, and stage 4 ERMs were defined as mild and thin and a foveal depression was present, associated with widening of the outer nuclear layer and loss of the foveal depression, associated with continuous ectopic inner foveal layers crossing the entire foveal area, and thick and associated with continuous ectopic inner foveal layers, respectively [10]. Eyes with additional retinal and macular pathology such as retinitis pigmentosa, Stargardt's disease, age-related macular degeneration, severe macular pathology (stage 4 ERM), degenerative myopia, central serous chorioretinopathy, diabetic retinopathy, diabetic macular edema, cystoid macular edema, macular schisis, or macular hole were excluded from the study. In addition, the cases with corneal or optic nerve related pathology were also excluded. Additional systemic diseases such as hypertension and diabetes mellitus were questioned in detail, and cases with diabetes mellitus were excluded from the study.

Cases included were divided into two groups. Group 1 (control group) and Group 2 (study group) were formed with cases with monofocal and multifocal IOL, respectively. Refractive errors and decentration levels of IOLs were noted before macular surgery. A Scheimpflug camera was used to evaluate decentration of IOL [11]. Refractive error was determined by using an autorefractometer with at least three similar results (Nidek ARK-530A, Gamagori, Japan). All patients underwent complete ophthalmologic examinations including evaluation of the refractive status, measurement of BCVA for distance, slit-lamp examination, indirect ophthalmoscopy, fundus fluorescein angiography (FFA), and optical coherence tomography (OCT) imaging performed preoperatively and postoperatively. The patients were followed up to the 6^th^ postoperative month.

All patients provided informed consent for sutureless 23-gauge PPV with ERM and internal limiting membrane (ILM) peeling assisted by triamcinolone acetonide and Brillant Blue G (BBG) dye under retrobulbar anesthesia. This study was approved by the University of Health Sciences Ethics Committees (Study number: 17073117-050.99-2785) and conducted according to the Declaration of Helsinki.

The ERM and ILM peeling process were performed by the same experienced vitreoretinal surgeon (AA) and device (Constellation, Alcon, USA). In all cases, a flush of 0.3 mL of triamcinolone acetonide was used to confirm the posterior hyaloid removal. Scleral indentation was performed for completing the removal of the vitreous base and identification of possible retinal breaks. In all cases, the macular region was stained with BBG dye. ERM and ILM peeling were performed together limited to the macular region in all patients, with the help of microforceps by holding ILM from just outer border of ERM. The time required for macular surgery, beginning with holding of ILM and ending with complete peeling of membranes, was measured with the aid of a stopwatch.

Distribution of variables was measured by the Kolmogorov–Smirnov test. The Mann–Whitney *U* test was used in the analysis of quantitative independent data. The Wilcoxon test was used to analyze dependent quantitative data. The Chi-square test was used in the analysis of qualitative independent data. Spearman correlation analysis was used in the correlation analysis. SPSS 26.0 program was used in the analysis, and the significance level (*P* value) was set at less than 0.05.

## 3. Results

A total of 70 eyes of 70 patients were included in the study. There were 55 and 15 cases in Group 1 and Group 2, respectively. All eyes in Group 2 had previously implanted multifocal IOL with the same properties (Panoptix, Alcon, USA). None of the eyes included in the study had corneal and iris-related pathologies in slit-lamp examination findings and no vitreous or retina-related problem other than ERM in indirect ophthalmoscopy, FFA, and OCT results. The mean age was 63.0 ± 4.6 and 60.3 ± 4.0 years, and the ratio of female:male was 38 : 17 and 10 : 5 in Group 1 and Group 2, respectively. There was no statistically significant difference between the groups in terms of age (*P* > 0.05) and gender (*P* > 0.05) (Table 1).

**Figure 1:**
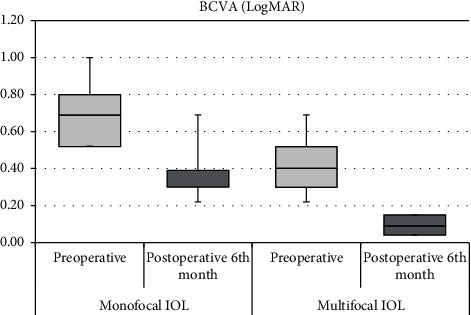
Best corrected visual acuities (BCVA) of groups before and after macular surgery.

Before macular surgery, the mean BCVA in Group 1 and Group 2 was 0.69 ± 0.15 and 0.38 ± 0.14 logMAR, respectively. The mean BCVA in Group 1 and Group 2 improved to 0.40 ± 0.14 and 0.10 ± 0.04 logMAR, respectively, at the 6^th^ month after PPV. BCVA at the 6^th^ month postoperatively improved in both groups compared to the preoperative period (*P* < 0.05). The mean BCVAs in Group 2 at the preoperative and postoperative 6^th^ month were statistically significantly higher than in Group 1 (*P* < 0.05) (Table 2) (Figure 1).

**Figure 2:**
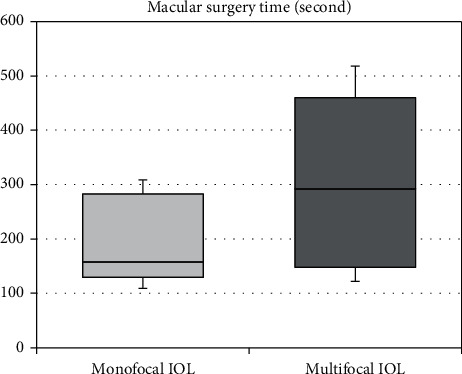
The time for macular surgery of groups.

The mean spherical refraction error was 0.56 ± 0.26 diopters in Group 1 and 0.52 ± 0.27 diopters in Group 2. The mean IOL decentration in Group 1 and Group 2 was 1.07 ± 1.23 mm and 0.93 ± 1.22 mm, respectively. There was no statistically significant difference between the groups in terms of the mean spherical refraction error (*P* > 0.05) and IOL decentration level (*P* > 0.055) (Figure 1). The mean time required for macular surgery in Group 2 was statistically significantly longer than Group 1 (*P* < 0.05) (Table 3) (Figure 2). The most important condition associated with prolongation of macular surgery was the level of decentration in multifocal IOL.

There was no statistically significant relationship between mean spherical refraction error and macular surgery time in both groups. There was no statistically significant relationship between IOL decentration and macular surgery time in Group 1 (*P* > 0.05), but it was found in Group 2 (*P* < 0.05). In Group 2, there was a positive correlation between IOL decentration and macular surgery time (Table 4).

Touch-related iatrogenic peripheral retinal breaks developed in 6 (8.5%) and 4 (26.6%) eyes in Group 1 and Group 2, respectively. The frequency of retinal breaks was not connected with decentration of multifocal IOLs. All retinal breaks were treated only with argon laser photocoagulation without the need for endotamponads intraoperatively.

## 4. Discussion

According to the World Health Organization data, cataract surgery is the most common surgical procedure in the world, and it is estimated that many people over the age of 65 will benefit from this operation in the next decade [12, 13]. Expectations from cataract surgery have increased in the recent years. In addition to the increase in the degree of vision, an improvement in the quality of vision has become an expectation. Cataract surgery has become an increasingly common operation as the development of intraocular lens technology meets these expectations [14]. The lens placed in the eye is vital in ensuring vision quality after cataract surgery. There are many studies in the literature proving that multifocal IOLs are more successful in providing independence from close vision and glasses than monofocals [15].

Some studies reporting that multifocal IOLs of multiple concentric optical regions with different dioptric power cause various optical limitations. Macular diseases are relative contraindications for multifocal IOL implantation [16]. Braga-Mele et al. reported development of dystopic disorders, glare, and decreased contrast sensitivity after multifocal IOL implantation in patients with macular disease [9]. In the literature, investigation of the results of PPV that was performed simultaneously or later in eyes undergoing cataract surgery has been published in some studies [17–19]. According to our knowledge and detailed literature research, there was no study investigating the effect of multifocal IOL on macular surgery. With this aspect, our study is unique.

Patel et al. reported increased the risk of retinal tears during the combined PPV and multifocal IOL implantation surgery procedure performed for symptomatic vitreous opacity, in their 5-case study [17]. In our study, iatrogenic retinal brakes occured more frequently (26.6%) in the eyes with multifocal IOL. According to our experience, we think this is due to fluctuations in the view of the surgeon. In another study, Patel et al. reported visually successful results after ERM peeling performed during the combined phacovitrectomy operation in the eyes with multifocal IOL implantation [18]. Navarro et al. prospectively analysed the results of PPV for symptomatic posterior vitreous detachment in the eyes of 16 patients who underwent multifocal IOL implantation after cataract surgery and reported that vitroretinal surgery significantly improved visual acuity and quality [19]. In our study, a statistically significant increase in BCVA developed in both groups at the 6^th^ month. The mean BCVA was higher in Group 2 than in Group 1 in both the preoperative and postoperative 6^th^ month. The mean distant BCVA difference between the two groups, found not only in the postoperative but also in the preoperative period, suggests that this result might be due to the loss of ERM-related macular function.

Visual quality achieved by the surgeon during macular surgery is usually one of the important factors affecting the operation time. Spherical refraction error could be easily corrected by imaging systems connected to the operating microscope. The results of our study support this hypothesis. Our results indicate that there was no statistically significant difference between the groups in terms of the mean spherical refraction error, and the most important parameter extending macular surgery was the decentration of multifocal IOL. Multifocal IOLs have rings with different refractive power. The angle of view obtained from the central area is usually sufficient for macular surgery. IOL decentration may cause the surgeon to look behind the narrow concentric region in the periphery rather than the large region of the multifocal IOL in the center and fluctuate the image. These sight fluctuations may cause the surgeon to hesitate while peeling membranes, try to restore the centralization of the eye to clarify the image, and ultimately spend more time. Advanced IOL decentration may also make it impossible for the surgeon to provide view from the same concentric region. In our study, intraoperative iatrogenic retinal breaks developed significantly more frequently in eyes with multifocal IOL. However, this complication was not related to IOL decentration. This may be due to the surgeon's vision usually being provided from the outer rings of multifocal IOLs during vitreous base cutting.

Small sample size and short follow-up time of the study group are the limitations of this study. More comprehensive long-term randomized trials are needed to evaluate the effects of IOL types on macular surgery. Another situation to be mentioned about our study is the difference between the number of cases that may influence comparison between groups.

## 5. Conclusions

In cases with multifocal IOL, macular surgery could be successfully completed. The most important conditions that could impair the vision clarity of the surgeon seem to be decentration of the multifocal IOL that might be related with surgical difficulty and higher risk of intraoperative complications.

## Figures and Tables

**Table 1:** Characteristic and demographic information of the groups.

		Monofocal IOL (Group 1)			Multifocal IOL (Group 2)			
		Mean ± SD/(*n* − %)		Median	Mean ± SD/(*n* − %)		Median	*P*
Age (years)		63.0 ± 4.6		63.0	60.3 ± 4.0		60.0	0.057m

Gender	Male	17	30.9%		5	33.3%		0.858*X*^2^
	Female	38	69.1%		10	66.7%		

**Table 2:** Best corrected best visual acuity of the groups.

	Monofocal IOL (Group 1)		Multifocal IOL (Group 2)		*P*
	Mean ± SD/(*n* − %)	Median	Mean ± SD/(*n* − %)	Median	
BCVA (LogMAR)					
Preoperative	0.69 ± 0.15	0.69	0.38 ± 0.14	0.39	0.000 m
Postoperative 6^th^ month	0.40 ± 0.14	0.40	0.10 ± 0.04	0.09	0.000 m
Preoperative-postoperative change	−0.29 ± 0.14	−0.30	−0.29 ± 0.14	−0.26	0.902 m
Intra-group change *P*	0.000^w^		0.001^w^		

m: Mann−WhitneyU-test; *X*^2^: chi-square test; w: Wilcoxon test; D: diopter; mm: millimeter; sec: second; BCVA: best corrected visual acuity.

**Table 3:** The mean refractive error, IOL decentration, and macular surgery of the groups.

	Monofocal IOL (group 1)		Multifocal IOL (group 2)		*P*
	Mean ± SD/(*n* − %)	Median	Mean ± SD/(*n* − %)	Median	
Spherical refraction error (D)	0.56 ± 0.26	0.50	0.52 ± 0.27	0.50	0.628 m
IOL decentration (mm)	1.07 ± 1.23	0.00	0.93 ± 1.22	0.00	0.622 m
Macular surgery time (sec)	191.5 ± 71.5	158.0	305.3 ± 157.3	292.0	0.013 m

m: Mann−WhitneyU-test; *X*^2^: chi-square test; w: Wilcoxon test; D: diopter; mm: millimeter; sec: second; BCVA: best corrected visual acuity.

**Table 4:** The relationship between mean spherical refraction error, IOL decentration, and macular surgery time.

	Macular surgery time in group 1		Macular surgery time in group 2	
	*r*	*P*	*r*	*P*
Spherical refraction error (D)	0.268	0.053	0.076	0.788
IOL decentration (mm)	−0.035	0.800	0.875	0.000

Spearman correlation test; D: diopter; mm: millimeter; IOL: intraocular lens.

## Data Availability

The datasets generated and analysed during the current study are not publicly available due to prohibition of our archive system but are available from the corresponding author on reasonable request.
